# Beating Thermal Deterioration of Magnetization with Mn_4_C and Exchange Bias in Mn–C Nanoparticles

**DOI:** 10.3390/nano8121056

**Published:** 2018-12-15

**Authors:** Ping-Zhan Si, Xin-You Wang, Hong-Liang Ge, Hui-Dong Qian, Jihoon Park, Yang Yang, Yin-Sheng Li, Chul-Jin Choi

**Affiliations:** 1 College of Materials Science and Engineering, China Jiliang University, Hangzhou 310018, China; wxy1003@163.com (X.-Y.W.); hongliang_ge@cjlu.edu.cn (H.-L.G.); 2 Functional Nanopowders Materials Department, Korea Institute of Materials Science, Changwon 51508, Korea; qianhuidong@kims.re.kr (H.-D.Q.); jpark@kims.re.kr (J.P.); yangyang@kims.re.kr (Y.Y.); 3 Engineering Ceramics Research Group, Korea Institute of Materials Science, Changwon 51508, Korea; derek.ys.li@hotmail.com

**Keywords:** exchange bias, nanoparticles, Mn_4_C, arc discharge

## Abstract

The magnetization of most materials decreases with increasing temperature due to thermal deterioration of magnetic ordering. Here, we show that Mn_4_C phase can compensate the magnetization loss due to thermal agitation. The Mn–C nanoparticles containing ferrimagnetic Mn_4_C and other Mn–C/Mn-O phases were prepared by using the traditional arc-discharge method. A positive temperature coefficient of magnetization (~0.0026 Am^2^ kg^−1^ K^−1^) and an exchange bias up to 0.05 T were observed in the samples. We ascribe the exchange bias to the co-existence of ferrimagnetic Mn_4_C/Mn_3_O_4_ and antiferromagnetic α-Mn(C)/MnO phases. The positive temperature coefficient of magnetization of the samples was ascribed to the presence of Mn_4_C phase, which is considered as a Néel’s *P*-type ferrimagnet.

## 1. Introduction

The magnetization of most magnetic materials decreases with increasing temperature due to thermal agitation of magnetic ordering. The thermal-induced magnetization reduction severely restricts the applications of magnetic materials at elevated temperatures. From a technical point of view, it is highly desired to find materials that can resist thermal deterioration of magnetization. Our previous work showed that the magnetization of high-purity Mn_4_C powders increases linearly with increasing temperature at temperatures up to 590 K [1]. The positive temperature coefficient of magnetization in Mn_4_C at temperatures above room temperature might be potentially useful in tuning the thermomagnetic behaviours of a magnetic system containing phases with a negative temperature coefficient of magnetization. However, no report in this field could be found. In this work, the Mn–C nanoparticles composed of phases with both positive and negative temperature coefficient of magnetization were prepared by arc discharge method. The unusual thermomagnetic behaviours of the Mn–C nanoparticles were studied.

The current understandings of the Mn–C system have evolved gradually since the systematic investigation of this system by Vogel and Döring in 1935 [2,3]. Although a number of high quality experimental and theoretical investigations have been conducted, there are still a number of controversial issues [1,2,3]. The constitutional diagram of Mn–C was proposed containing five stoichiometric carbides (Mn_23_C_6_, Mn_7_C_2_, Mn_3_C, Mn_5_C_2_, and Mn_7_C_3_) and five solid solution phases (α-, β-, γ-, δ-, and ε-Mn) [3,4,5]. However, Zaitsev’s results do not support the existence of a carbide at a carbon concentration of 22.2 at.%, referred to as Mn_7_C_2_ by Kuo and Persson [4,6]. The Mn_4_C phase reported by Morgan in 1954 was soon suggested to be Mn_4_(C, O) and Mn_4_C was thought to be unstable at room temperature [4,7,8]. However, high-purity Mn_4_C powders were prepared successfully recently [1]. Moreover, fairly large uncertainties remain for the phase equilibria and thermodynamic properties of the Mn–C solid solution phases [3]. The controversial issues on stoichiometric manganese carbides and the substantial uncertainties concerning the Mn–C solid-state phase equilibria of the solid solution phases indicate the need for a more detailed study on the Mn–C system. Most of the previous work on Mn–C system has been focused on bulk samples. It has been proved that a low-dimensional system usually exhibits unique structure and properties that differ from that of the bulk counterpart due to size effect and surface effect [9]. In this work, the structure and magnetic properties of Mn–C nanoparticles prepared by arc discharge method were studied.

The exchange bias effect has been extensively studied in exchange-coupled nanostructures with ferromagnetic (FM) and antiferromagnetic (AFM) interfaces. The macroscopic signature of this interfacial exchange interaction is a shift in the magnetic hysteresis loop along the magnetic field axis. A variety of exchange bias systems such as Ni/Ni(OH)_2_, MnN/CoFe, CoO/Co, LaMnO_3_/LaNiO_3_, Mn_3_O_4_/MnO/Mn, and Gd/Cr, etc., have been studied experimentally and/or theoretically [10,11,12,13,14,15,16,17,18]. In this work, a novel exchange bias Mn–C system that containing ferrimagnetic Mn_4_C, AFM α-Mn(C), manganese oxide, and trace amount of Mn_3_O_4_ were prepared and studied.

## 2. Materials and Methods

The Mn–C nanoparticles were prepared by the traditional arc-discharge method in argon atmosphere (0.03 MPa). A Mn–C alloy in nominal composition of Mn_4_C was used as anode, while a tungsten needle served as cathode. Argon plasma was struck between the two electrodes and maintained for ten minutes. The powder deposited on the water-cooled chamber was passivated in the chamber for 10 h, and then collected in air and separated by using a hand magnet. The structure of the magnetic powder was measured by using X-ray powder diffraction (XRD, Rigaku D/Max 2500, Tokyo, Japan) with Cu Kα radiation. The morphology of the magnetic powder was observed by using transmission electron microscopy (TEM, Jeol 200CX, Tokyo, Japan). The magnetic properties of the samples were measured using a physical properties measurement system (Quantum Design Inc., San Diego, CA, USA).

## 3. Results and Discussion

### 3.1. Structure

**Figure 1:**
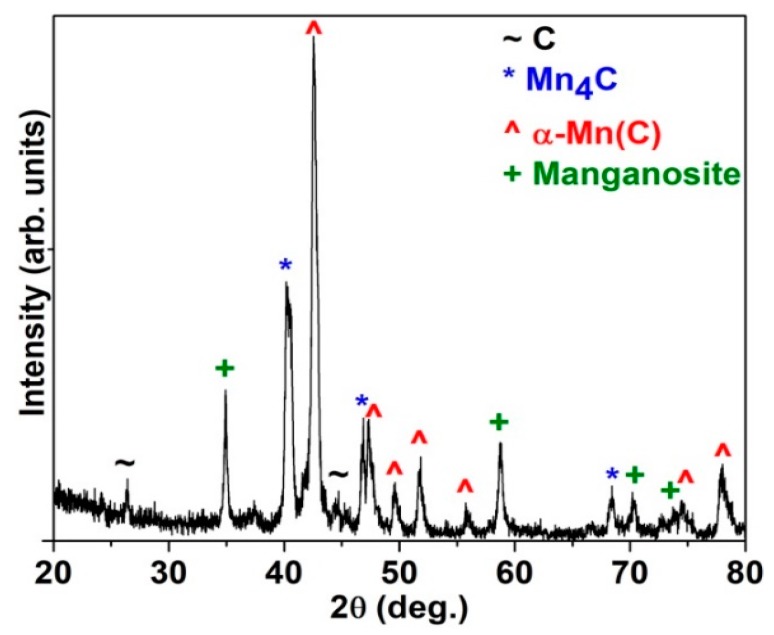
The XRD patterns of Mn–C nanoparticles can be indexed with α-Mn(C), Mn_4_C, manganosite, and trace amount of C.

The XRD patterns of the Mn–C nanoparticles, as shown in Figure 1, could be indexed mainly with α-Mn(C) solid solution, Mn_4_C, manganosite, and trace amount of C. According to the Mn–C phase diagram [3], the α-Mn(C) solid solution is the most stable phase at temperatures below 1000 K in the Mn-rich region. The carbon solution in manganese metal is usually described by an interstitial solution model with one sublattice occupied by manganese atoms and the other occupied by carbon and vacancies [3]. The interstitial C atoms may significantly enlarge the lattice parameters of α-Mn. The diffraction peaks of α-Mn(C) shift slightly to lower angles in comparison with that of pure α-Mn, indicating enlarged lattice parameters due to interstitial C. The formation of the manganosite was ascribed to the spontaneous oxidation of Mn nanoparticles when exposed to air, and this had been observed in pure Mn nanoparticles with large surface areas [9]. The trace amount of C may result from two routes, the unreacted C and the C that precipitated from the Mn–C melt when cooling down. Although the Mn_4_C phase is thermally unstable and thus is absent in the Mn–C phase diagram [3], a considerable amount of Mn_4_C phase was detected by XRD, as shown in Figure 1. The formation mechanism of Mn_4_C phase is not clear. The room temperature ferromagnetic behaviours of the Mn–C nanoparticles as discussed below are further evidence for the presence of Mn_4_C in the samples as most other phases are AFM or paramagnetic (PM) at room temperature.

### 3.2. Morphology

**Figure 2:**
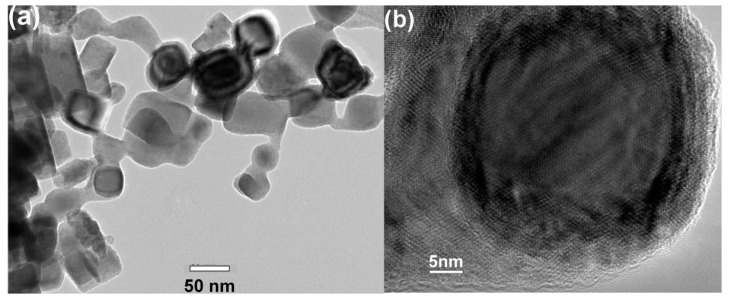
The low magnification (**a**) and high magnification (**b**) TEM images of the Mn–C nanoparticles. A shell/core structure of the Mn-C nanoparticles was observed.

Figure 2 shows the morphology of the Mn–C nanoparticles observed by the TEM. Most nanoparticles show cubic or spherical shape with size of approximately several tens of nanometres. The size distribution of these nanoparticles is relatively large, as shown in Figure 2a. A high-resolution image of a typical nanoparticle is shown in Figure 2b, which shows that the surface layer of the nanoparticle is different from the cores. The thickness of the surface layer is approximately 3–5 nm while the diameter of the core is approximately 30 nm. Such shell/core structure is mainly attributed to the oxidation process of very small Mn-C nanoparticles when exposed to air. In fact, an analogous shell/core structure has been observed in pure Mn nanoparticles [9]. It is reasonable for us to assume that most manganosite as observed by XRD present as the surface shell.

### 3.3. Magnetic Properties

**Figure 3:**
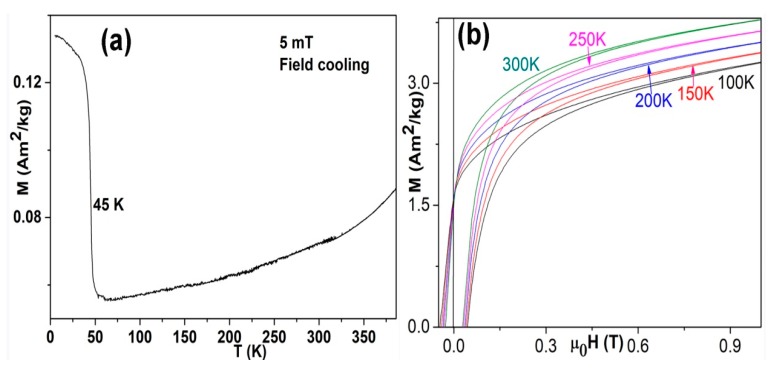
(**a**) The M-T curve of the field-cooled Mn–C nanoparticles measured with increasing temperature and under an applied field of 5 mT. (**b**) The magnetic hysteresis loops (quadrants 1 and 2) of the Mn–C nanoparticles at 100 K, 150 K, 200 K, 250 K, and 300 K, respectively.

Figure 3a shows the M-T plot of the Mn–C nanoparticles measured with increasing temperature after cooling to 5 K from 300 K under an applied field of 5 mT. The magnetization of the nanoparticles decreases abruptly at ~45 K, which is very close to the Curie temperature (~43 K) of Mn_3_O_4_. Our previous work on Mn nanoparticles indicated that Mn_3_O_4_ phase is usually spontaneously formed when the Mn nanoparticles were exposed to air [9]. Although Mn_3_O_4_ were not detected by XRD, as shown in Figure 1, we could not exclude its presence for the following reasons. First, the presence of manganosite has been proved by XRD as seen in Figure 1. The oxygen stoichiometry in the manganosite formed via spontaneous passivation of the nanoparticles is difficult to measure. However, it is reasonable for us to assume a deceasing oxygen concentration from the surface to the centre of the Mn–C nanoparticles, as partially proved by the shell/core structure of the nanoparticles shown in Figure 2b. Second, the low fraction and the tiny grain size of Mn_3_O_4_ make it difficult to be detected by XRD for the limited sensitivity of XRD. Third, only Mn_4_C and Mn_3_O_4_ are ferrimagnetic (FI) phases among Mn–C and Mn-O binary compounds while all the other manganese carbides and manganese oxides are AFM or PM at low temperatures. Our recent work showed that the magnetization of the Mn_4_C phase varies little with temperatures below 50 K [1]. Therefore, the large magnetization variation at ~45 K was ascribed to the FI/PM transition of Mn_3_O_4_. However, the abrupt magnetization change and positive temperature coefficient of magnetization observed here were not obviously observed in the Mn_4_C/MnO micro-powders prepared by laser-ablation method [19]. We speculate that the dimension of the powder and the fraction of Mn_4_C in the powder may play an important role in phase formation and thermomagnetic behaviours of the sample.

It is interesting that the magnetization of our Mn–C nanoparticles increases with increasing temperature at temperatures above 60 K. Most materials exhibit a monotonic decrease of magnetization with increasing temperature due to thermal agitation. Our previous work showed that the magnetization of the Mn_4_C phase increases with increasing temperature at temperatures above ~50 K [1]. Therefore, we ascribe the positive temperature coefficient of magnetization in the Mn–C nanoparticles to the presence of Mn_4_C phase. The presence of other phases with negative temperature coefficients of magnetization may reduce the magnetization increment rate of the samples, giving an opportunity to control the thermodynamics of the magnetization of the magnetic material, and this has been proved by the M-H curves (Figure 3b) of the samples.

Figure 3b shows the magnetic hysteresis loops of the Mn–C nanoparticles at varied temperatures. The magnetization (at 1 T) of the Mn–C nanoparticles at 100 K, 150 K, 200 K, 250 K, and 300 K is 3.26 Am^2^ kg^−1^, 3.38 Am^2^ kg^−1^, 3.51 Am^2^ kg^−1^, 3.64 Am^2^ kg^−1^, and 3.78 Am^2^ kg^−1^, respectively. The monotonic increase of the magnetization with increasing temperature was ascribed to Mn_4_C. It is known that the ferrimagnetic Mn_4_C has a simple cubic perovskite-type structure and has two ferromagnetic sublattices that composed of face-centred Mn atoms and cornered Mn atoms, respectively [1]. The positive temperature coefficient of Mn_4_C can be explained by Néel’s *P*-type ferrimagnetism, which appears when the sublattice with smaller moment is thermally disturbed more easily [1,20]. The temperature coefficient of magnetization of Mn–C nanoparticles is approximately 0.0026 Am^2^ kg^−1^ K^−1^, which is lower than that (~0.0072 Am^2^ kg^−1^ K^−1^) of the pure Mn_4_C [1], owing to the presence of other phases with negative temperature coefficients of magnetizations. This work indicates that the magnetization of a system composed of materials with both positive and negative temperature coefficients of magnetizations may be tunable by tuning the fraction of these two component phases. The remnant magnetization of the Mn–C nanoparticles varies little with temperature and is approximately 1.58 Am^2^/kg. The coercivity of the Mn–C nanoparticles falls in the range of 0.046–0.026 T in the temperature range of 100–300 K, as shown in Figure 3b.

**Figure 4:**
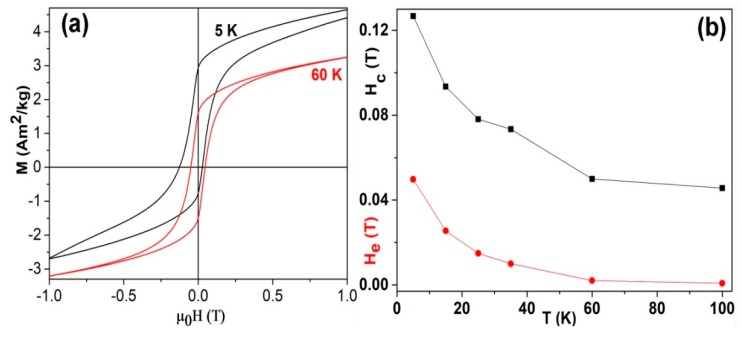
(**a**) The typical field-cooling (1 T) M-H curves and (**b**) the temperature dependence of exchange bias (H_e_) and coercivity (H_c_) of the Mn–C nanoparticles.

Figure 4 shows the typical M-H curves and the temperature dependence of exchange bias (H_e_) and coercivity (H_c_) of the Mn–C nanoparticles. Figure 4a shows an obvious shift of the magnetic hysteresis loops along the axis, indicating exchange bias effect in the samples. We ascribe the exchange bias to the co-existence of ferrimagnetic Mn_4_C/Mn_3_O_4_ and antiferromagnetic α-Mn(C)/MnO phases. An applied field of 1 T is enough to saturate the sample at temperatures above 60 K, as seen in Figure 3b and Figure 4a, but is not enough to saturate the sample at 5 K, owing to the magnetic contribution of Mn_3_O_4_ phase at temperatures lower than its Curie point (43 K). Our previous work on Mn_4_C and Mn_3_O_4_ showed that Mn_4_C can be saturated under much lower magnetic field in comparison with Mn_3_O_4_ at low temperatures near 5 K [1,12]. The H_e_ and H_c_ of the Mn–C nanoparticles at 5 K reached up to 0.05 T and 0.13 T, respectively. Both H_e_ and H_c_ decrease with increasing temperature, as shown in Figure 4b. The values of H_e_ and H_c_ are much lower than that of the oxide-coated Mn nanoparticles prepared by the same method [9,12]. indicating a reduced interfacial exchange coupling between the FI and AFM phases in comparison with that of the oxide-coated Mn nanoparticles. The exchange bias effect in the Mn–C nanoparticles may result from the interfacial coupling between AFM α-Mn(C) solid solution or manganosite and FI Mn_3_O_4_ or Mn_4_C, respectively. We speculate that the interstitial C atoms in the α-Mn lattices might reduce the antiferromagnetic coupling of the Mn atoms and thus the interfacial coupling for exchange bias effect. 

## 4. Conclusions

The Mn–C nanoparticles composed of α-Mn(C) solid solution, Mn_4_C, manganosite, and trace amounts of C/Mn_3_O_4_ were prepared by the traditional arc discharge method. The Mn–C nanoparticles exhibit room temperature ferromagnetic behaviours and unusual positive temperature coefficients of magnetization owing to the presence of Mn_4_C, which is Néel’s *P*-type ferrimagnet. The thermomagnetic behaviours of a system composed of materials with both positive and negative coefficients of magnetizations may be tunable by tuning the fraction of the component materials. The exchange bias field and the coercivity of the Mn–C nanoparticles decrease with increasing temperature.
